# An isothermal CRISPR-based diagnostic assay for *Neisseria gonorrhoeae* and *Chlamydia trachomatis* detection

**DOI:** 10.1128/spectrum.00464-23

**Published:** 2023-10-26

**Authors:** Hao Luo, Lihong Zeng, Xiaona Yin, Yuying Pan, Jianjiang Yang, Mingjing Liu, Xiaolin Qin, Zhanqin Feng, Wentao Chen, Heping Zheng

**Affiliations:** 1 Dermatology Hosptial, Southern Medical University, Guangzhou, China; 2 Guangzhou Key Laboratory for Sexually Transmitted Diseases Control, Guangzhou, China; 3 Medical College, China Three Gorges University, Yichang, China; Tainan Hospital, Ministry of Health and Welfare, Tainan, Taiwan

**Keywords:** *Neisseria gonorrhoeae*, *Chlamydia trachomatis*, coinfection, CRISPR‒Cas12a, CRISPR‒Cas13a, dual-target detection

## Abstract

The occurrence of *Chlamydia trachomatis* (CT) and *Neisseria gonorrhoeae* (NG) is increasing worldwide, particularly in low- and middle-income countries. In cases where most infections are asymptomatic and remain in a coinfection condition. It may cause missed detections or the severe subsequent symptoms. Accurate diagnosis and convenient screening are essential for disease control. However, molecular diagnostics for CT/NG predominantly rely on polymerase chain reaction (PCR)-based detection, which requires special devices and conditions. Here, we developed an isothermal CRISPR-based CT/NG dual-target detection system by pairing recombinase polymerase amplification (RPA) and CRISPR-Cas12a/13a detection. Multiplex RPA can amplify NG and CT simultaneously, and a single-pot reaction with the Cas12a/13a can be performed for further recognizing the amplified target, driving the cleavage of the corresponding fluorescence reporter separately; thus, the signal can be monitored in different channels. This CRISPR-based CT/NG detection system achieved analytical sensitivities of 10° copies/μL for both synthetic CT/NG dsDNA and exhibited no cross-reaction with other species. In tests on 88 clinical samples, our CRISPR-based assay showed excellent agreement with the commercial assay Roche Cobas 4800 (100% accuracy for NG, 94.32% accuracy for CT, and 97.73% accuracy for CT/NG coinfection) and showed higher positive percent agreement, negative percent agreement, and accuracy than those reported for TaqMan PCR in a previous study. In addition, the results of this CRISPR-based system could be acquired within 75 min. Our isothermal diagnostic method with promising performance provides more convenience than PCR-based methods for detection and screening.

**IMPORTANCE:**

A method for *Neisseria gonorrhoeae* (NG)/*Chlamydia trachomatis* (CT) detection is developed using multiplex-recombinase polymerase amplification and Cas12a/Cas13a. This method can detect NG and CT simultaneously with high sensitivity and specificity. This method has great potential to be further developed into larger-scale screening and point-of-care testing (POCT).

## INTRODUCTION

Sexually transmitted infections (STIs), such as *Neisseria gonorrhoeae* (NG) and *Chlamydia trachomatis* (CT) infection, have imposed a high morbidity worldwide. In 2021, the World Health Organization estimated that there were 82 million cases of gonorrhoea (caused by NG) and 128 million cases of chlamydia (caused by CT) worldwide (1). Individuals with chlamydia and women with gonorrhoeae are commonly asymptomatic; moreover, studies have reported that NG and CT coinfection is frequent (2 – 6). Without timely and effective therapy, urogenital infections with NG/CT cause serious reproductive tract complications, such as pelvic inflammatory disease, ectopic pregnancy and infertility (7 – 9), an increased risk of human immunodeficiency virus acquisition (10), and neonatal blindness (11). Therefore, the US Preventive Services Task Force recommended routine chlamydia screening of young, sexually active women in 1989; similarly, in 2003, the UK Health Security Agency launched a national chlamydia screening program for sexually active women and men under 25 years of age (12, 13).

The traditional culture-based assay is regarded as the gold standard for detecting NG and CT, but its complex procedure and long turnaround time hinder its application in large-scale routine screening, especially at primary care facilities (14, 15). Currently, nucleic acid amplification tests (NAATs), which are highly sensitive and specific as well as time-saving, are recommended in the detection of NG and CT by the American Centers for Disease Control and Prevention (CDC) and European CDC (16, 17). The performance of NAATs for the detection of NG and CT has been reported, with 100% sensitivity for both CT and NG by Aptima Combo2 and Amplicor PCR, and approximately 80% sensitivity with ProbeTec strand displacement amplification (SDA) (18). However, these NAATs are heavily reliant upon special instruments and strict testing environments, which remain inaccessible in many resource-limited regions (e.g., low- and middle-income countries, rural and remote regions) (19). The isothermal amplification technique is an alternative NAAT that does not need a special thermocycler and is thus more convenient for detection and screening. Several isothermal amplification techniques have been established for NG and CT detection. For example, transcription-mediated amplification (TMA) targeting RNA has been utilized in NG and CT detection; however, this method requires a special instrument and skilled technicians (20). Loop-mediated isothermal amplification (LAMP) was created by Notomi et al. (21); the use of this method for NG and CT detection was designed but not experimentally confirmed (22). Recombinase polymerase amplification (RPA) is another newly developed isothermal amplification assay, and RPA-based dual-target amplification of NG and CT has been developed and evaluated with lateral flow strips, showing a limit of detection (LOD) of 200 plasmid copies per reaction (23), but RPA can lead to nonspecific amplification (24). Overall, the development of rapid, highly sensitive, highly specific, and convenient dual-target (NG and CT) detection assays for routine STI screening should be considered a priority (25).

Clustered regularly interspaced short palindromic repeat (CRISPR)-based diagnostic assays provide robust performance in the diagnosis of infectious diseases (e.g., Zika virus and *Treponema pallidum*), presenting the advantages of single molecular detection and single-base resolution (26, 27). Diagnosis by pairing isothermal amplification with CRISPR-based detection provides a means to overcome the disadvantages of existing methods for detecting NG/CT (e.g., our assay seeks to address challenges such as limited resources in certain regions, ease of use, and improved sensitivity and specificity, consistent with previous studies in this area). In this study, we developed and validated an isothermal assay to detect NG and CT by combining multiplex RPA and a dual-target CRISPR detection system (the mixture contains Cas12a and Cas13a). The whole process takes place at 37°C, which allows the use of the established novel detection system in a convenient way; moreover, this system addresses the first step of point-of-care testing (POCT) and holds great promise for further development to finally realize POCT, which provides timely detection that can reduce the risk of pathogen transmission (28).

## MATERIALS AND METHODS

### Clinical specimens

This study was approved by the Ethics Review Committee at the Dermatology Hospital of Southern Medical University (GDDHLS-2020056, 2021071). A panel of 12 genital microorganisms (8 common species including *T. pallidum*, *Herpes simplex virus-2*, *Candida albicans*, *Trichomonas vaginalis*, *Ureaplasma urealyticum*, *Mycoplasma humanum*, *Escherichia coli*, and *Enterococcus faecalis*, and 4 standard *Neisseria* species including *Neisseria meningitidis serogroup A*, *Neisseria meningitidis serogroup B*, *Neisseria meningitidis serogroup C*, and *Neisseria catarrhal*) were used to validate the analytical specificity of the CRISPR-based dual-target detection system. Eighty-eight urine clinical specimens, including 20 NG positive(+) samples, 20 CT positive(+) samples, 13 NG positive(+) and CT positive(+) coinfected samples and 35 CT negative(−) and NG negative(−) samples, and 12 swab samples, including 1 NG positive(+) sample, 1 CT positive(+) sample and 10 CT negative(−) and NG negative(−) samples, which were confirmed by Roche Cobas 4800 assay which were used to test the performance of our assay.

### The workflow of the CRISPR-based detection assay

**Fig 1:**
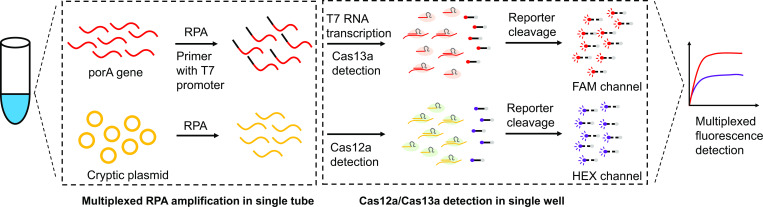
Schematic of the CRISPR-based dual-target detection system for NG and CT. The target genes of *N. gonorrhoeae* and *C. trachomatis* are the *porA* gene and cryptic plasmid, respectively, which are preamplified by multiplex RPA in a single tube with extracted DNA as the input. The RPA products are then transferred to the reaction mixture in a single well containing T7 RNA polymerase, Cas13a, Cas12a, target-specific crRNA, and two types of fluorescent reporter (FAM and HEX) for detection. FAM and HEX fluorescence channels monitor the results of Cas12a/Cas13a detection simultaneously.

We established an isothermal detection system for NG and CT by combining RPA and CRISPR‒Cas12a/Cas13a detection (Fig. 1). We selected the porA gene of NG and the cryptic plasmid of CT as targets; these targets have been widely used in previous studies (29, 30). Briefly, the targets were amplified by multiplex RPA reaction with primers (Table S1) and transferred to a CRISPR‒Cas12a/Cas13a detection system with optimized reaction criteria. Activated CRISPR‒Cas12a and Cas13a harbor collateral cleavage activity with ssDNA and RNA, respectively (31, 32), providing the ability to recognize dual reporters in a single detection. Synthesized RNA attached to the FAM reporter and ssDNA attached to the HEX reporter were employed for the detection of NG and CT, respectively.

### Screening of optimal RPA primers

All RPA primers used in this study are listed in Table S1. RPA primers were designed according to the National Center for Biotechnology Information Primer-Basic Local Alignment Search Tool with modified parameters [primer size: 30–35 nucleotide (nt)] and SnapGene (v4.1.9) and synthesized by Sangon Biotech (33). Specifically, the RPA primers for NG detection were appended with the T7 promoter sequence in the forward primers for further transcription. We selected the effective primers by cross-pairing the F1–F5 forward primers with the R1–R5 reverse primers in the RPA TwistAmp Basic kit (TwistDx, Berkshire, UK; TABAS03KIT). The total reaction volume (50 µL) contained 2.4 µL of each 10 µM primer, 29.5 µL of rehydration buffer, 2.5 µL of 280 mM magnesium acetate, 12.2 µL of double-distilled water (ddH_2_O), and 1 µL of DNA input. The samples were incubated at 37°C for 1 h, and Universal DNA purification kit (TIANGEN, Beijing, China; DP214) was used to purify the RPA products. The RPA products were analyzed by electrophoresis in a 2.0% agarose gel and visualized by UV transillumination.

### Preparation of crRNAs

crRNA was prepared according to previous reports (33, 34). Briefly, crRNAs for Cas12a and Cas13a detection were designed using SnapGene (v4.1.9) and synthesized by Sangon Biotech. Both Cas12a and Cas13a crRNAs contain the T7 promoter sequence, spacers (complementary to target, 20-nt sequence for Cas12a crRNA and 28-nt spacer sequence for Cas13a crRNA), and the core sequence (binding to Cas protein). Synthesized oligonucleotides (100 µM) were annealed to the short T7 promoter (100 µM) by a gradient annealing procedure. The product was then incubated at 37°C for 16 h using the HiScribe T7 Quick High Yield RNA Synthesis kit (New England Biolabs, MA, USA; E2050S) for crRNA transcription. RNA XP clean beads (Beckman, Brea, CA; A63987) were used to purify products and yield qualified crRNA. All crRNAs used in this study are shown in Table S1.

### Preparation of double-stranded DNA template

Two sets of primers (listed in Table S1) were used to construct the dsDNA template and further to evaluate the LOD of this assay. The first primer sets (NG-T7dsDNA1 and CT-dsDNA1) were used to screen for the optimal crRNAs and test the LOD of Cas12a or Cas13a. dsDNA was prepared using Q5 High-Fidelity DNA Polymerases (New England Biolabs, MA, USA; M0491L) to amplify the target in a 50-µL solution (10 µL of 5× Q5 Reaction Buffer, 10 µL of 5× Q5 High GC Enhancer, 1 µL of 10 mM dNTPs, 0.5 µL of Q5 DNA Polymerase, 2.5 µL of each 10 µM primer, 2 µL of DNA template, and 21.5 µL of ddH_2_O). PCR was run at 98°C for 30 s, then 40 cycles of 98°C for 10 s, 65°C for 30 s, and 72°C for 20 s, followed by 72°C for 2 min. PCR products were purified by a Universal DNA purification Kit (TIANGEN, Beijing, China; DP214) according to the manufacturer’s protocol, quantified with a Qubit dsDNA HS Assay Kit (ThermoFisher, MA, USA; Q33231), and stored at −20°C. The second primer set (NG-dsDNA2 and CT-dsDNA2) was used to test the LOD of the CRISPR-based multiplex detection assay. The procedures of PCR, DNA purification, and qualification were the same as those for the first primer set (except that 55°C was used as the annealing temperature). The amplified dsDNA was serially diluted 10 times and stored at −20°C.

### Establishment of the CRISPR-based dual-target assay

The CRISPR-based dual-target detection assay consisted of multiplex RPA and Cas12a/Cas13a collateral detection. The optimal RPA primers and crRNAs were selected as described above. Multiplex RPA was carried out using a TwistAmp Basic Kit (TwistDx, Maidenhead, Berkshire, UK; TABAS03KIT) according to the manufacturer’s instructions. The multiplex RPA reaction mixture consisted of 0.6 µL of each 10 µM primer, 14.75 µL of rehydration buffer, and 1.25 µL of 280 mM magnesium acetate, for a total volume of 25 µL. The DNA input of this assay was 1 µL per reaction, except that 5 µL was used for clinical specimen validation. RPA was performed at 37°C for 1 h (unless otherwise stated, 1 h was used throughout), and then 1.25 µL of RPA product was transferred for Cas12a/Cas13a collateral detection.

To establish the multiplex detection assay, we selected a reaction buffer according to the work of Li et al. (35). LbCas12a and LwCas13a were adapted for target recognition. Then, 25 µL reaction mixture contained 2.5 µL of CutSmart Buffer (New England Biolabs, MA, USA; B7204), 1 µL of rNTPs Mix (New England Biolabs, MA, USA; N0466 L), 1.25 µL of Murine RNase inhibitor (New England Biolabs, MA, USA; M0314 L), 0.75 µL of T7 RNA Polymerase (New England Biolabs, MA, USA; M0251 L), 200 nM Cas12a/Cas13a crRNA, 50 nM purified LbCas12a (KX-protein, Beijing, China; KX-E-002), 50 nM LwCas13a (Biolifesci, Guangzhou, China; M20202-0500), and 200 nM collateral RNA reporter and single-stranded DNA (ssDNA) reporter synthesized by Sangon Biotech. The reaction mixture was incubated for 1 h at 37°C on a 96-well half-area microplate (Corning, New York, USA; CLS3694-100EA) with fluorescent kinetics (Thermo Fisher, MA, USA; VL0000D0) measured at *Ex*/*Em* = 490 nm/520 nm for the FAM channel and at *Ex*/*Em* = 533 nm/559 nm for the HEX channel every 5 min. The RNA and ssDNA reporter are described in Table S1.

### DNA extraction of clinical urine samples

To evaluate the performance of the CRISPR-based dual-target assay in clinical samples, we collected 88 urine samples. DNA was automatically extracted from these samples by a Roche X480 (Roche Cobas 4800 system) and stored at −20°C.

### TaqMan PCR

TaqMan PCR was performed to validate the samples of eight genital microorganisms described above with the relevant probe and compared with the CRISPR-based dual-target assay in the detection of clinical urine samples. Primers and probes are available in Table S1 and were synthesized by Sangon Biotech. TaqMan PCR was performed using a 25-µL total reaction solution, including 12.5 µL of TaqMan Gene Expression Master Mix (Thermo Fisher Scientific, MA, USA; 4369016), 1.25 µL of 10 µM primers, 2 µL of 10 µM probe, 2 µL of 25 mM MgCl_2_, DNA template (input volume consistent with the CRISPR-based assay), and various amounts of ddH_2_O. The fluorescence value was measured on a Real-Time PCR Instrument with the following PCR procedure: 95°C for 10 min, 40 cycles of 95°C for 15 s, and 60°C for 1 min.

### Analysis of fluorescence data

Data are presented as mean ± SEM. Mean differences in quantification are determined by Student’s *t* test and were considered significant at *P* values < 0.05; ns, not significant; *, *P* < 0.05; **, *P* < 0.01; ***, *P* < 0.001; ****; *P* < 0.0001. The positive percent agreement (PPA) and negative percent agreement (NPA) represent the estimates of sensitivity and specificity, respectively. The PPA was calculated as true positives/(true positives + false negatives) × 100%, and the NPA was calculated as true negatives/(true negatives + false positives) × 100%. The overall percent agreement (accuracy) was calculated as (true positives + true negatives)/(true positives + false negatives + true negatives + false positives) × 100%. The PPA, NPA, accuracy, and κ value were calculated in MedCalc (version 11.4.2.0). Prism 8 software (GraphPad, La Jolla, CA, USA) was used for the visualization of the results and data analyses.

## RESULTS

### Optimization of CRISPR-based detection

**Fig 2:**
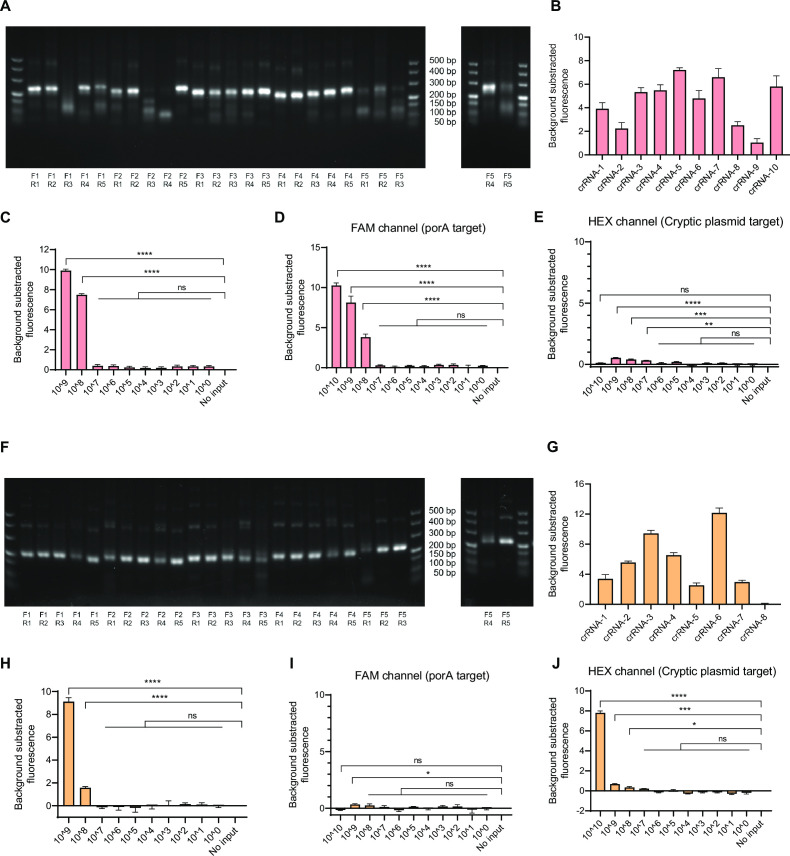
Establishment of the CRISPR-based dual-target detection assay. (**A**) Screening of optimal RPA primers for NG detection, and the five forward RPA primers appended the T7 promoter pair with five reverse RPA primers. Agarose gel electrophoresis was used to show the amplification results. (**B**) Screening of the optimal crRNA for Cas13a in NG detection. (**C**) Evaluation of the sensitivity of selected Cas13a crRNA in the Cas13a detection system with dsDNA template. (**D and E**) Evaluation of the sensitivity of selected Cas13a crRNA in the Cas12a/Cas13a dual-target detection system with the dsDNA template and measurement of the fluorescence value at the FAM and HEX channels. (**F**) Screening of optimal RPA primers for CT detection and five forward RPA primer pairs with five reverse RPA primers. Agarose gel electrophoresis was used to show the amplification results. (**G**) Screening of the optimal crRNA for Cas12a in CT detection. (**H**) Evaluation of the sensitivity of selected Cas12a crRNA in the Cas12a detection system with a dsDNA template. (**I and J**) Evaluation of the sensitivity of selected Cas12a crRNA in the Cas12a/Cas13a dual-target detection system with a dsDNA template and measurement of the fluorescence value at the FAM and HEX channels.

To optimize the reaction criteria of CRISPR-based Cas12a/Cas13a detection, we first screened the optimal RPA primers and crRNA sequence. The primer set (F3R5) exhibited the best performance for porA amplification after primer orthogonal tests (Fig. 2A). The sequence of crRNA-5 produced a higher fluorescence value than other crRNAs (Fig. 2B) and was selected for further sensitivity analysis. To evaluate the performance of Cas13a detection individually, synthetic dsDNA with an appended T7 promoter sequence was serially diluted. Cas13a detection with crRNA-5 achieved an NG-detection sensitivity of 10^8^ copies/μL under the FAM channel (Fig. 2C) with negligible background fluorescence under the HEX channel (Fig. 2D and E). For optimization of Cas12a detection, we selected the primer set (F5R3) for cryptic plasmid target amplification (Fig. 2F) and crRNA-6 for further testing (Fig. 2G). Without amplification of the target, crRNA-6 exhibited a CT-detection sensitivity of 10^8^ copies/μL under the HEX channel (Fig. 2H through J) with negligible background fluorescence under the FAM channel (Fig. 2I). Therefore, we successfully screened the optimal RPA primers and crRNA for the amplification system and Cas12a/Cas13a detection.

**Fig 3:**
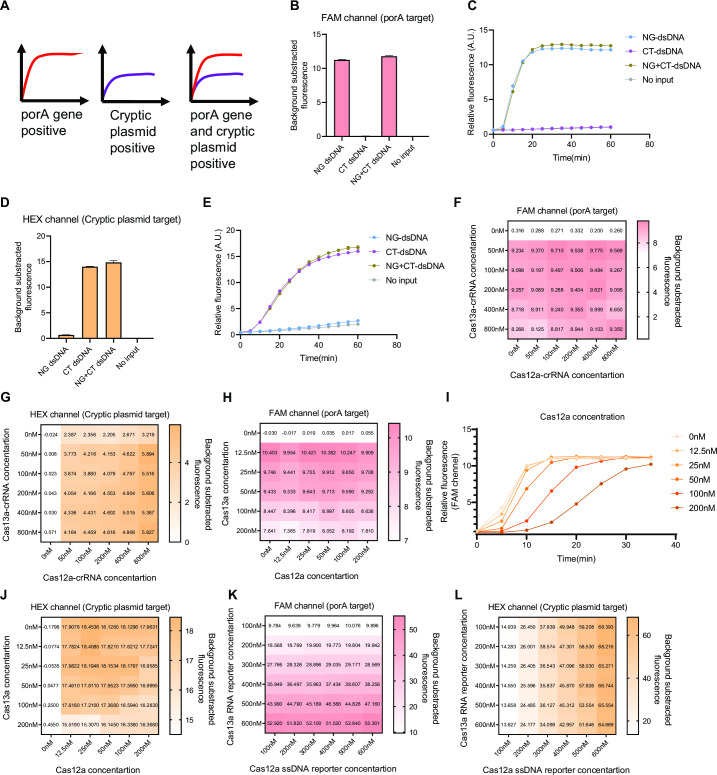
Evaluation of the effectiveness of various components of the CRISPR-based dual-target detection assay. (**A**) Schematic of the CRISPR-based dual-target detection, including two-channel monitoring. (**B and C**) Fluorescence value of the CRISPR-based dual-target detection assay in the FAM channel for detection of the NG and CT dsDNA templates. (**D and E**) Fluorescence value of the assay in the HEX channel for the detection of the NG and CT dsDNA templates. (**F and G**) Impact of various concentrations of Cas13a crRNA and Cas12a crRNA on the Cas12a/Cas13a detection system, assessed in the FAM and HEX channels. (**H**) Impact of various concentrations of Cas13a protein and Cas12a protein on the Cas12a/Cas13a detection system, measured at the FAM channel. (**I**) Increased concentrations of Cas12a protein reduce the effectiveness of Cas13a detection and the fluorescence values of Cas13a system are measured in FAM channel. (**J**) Impact of various concentrations of Cas13a protein and Cas12a protein on the Cas12a/Cas13a detection system, measured at the HEX channel. (**K and L**) Impact of various concentrations of reporters (FAM and HEX) on the Cas12a/Cas13a detection system, measured at the FAM and HEX channels.

We tested the performance of multiplex RPA (porA gene primers mixed with cryptic plasmid primers), and two sets of primers worked well independently (Fig. S1). Furthermore, we integrated selected primers and crRNAs to assemble a CRISPR-based dual-target detection system (Fig. 3A). We then used purified dsDNA to test our system. Both individual and mixed porA and cryptic plasmid dsDNA were successfully detected in 60 min (Fig. 3B through E). To optimize CRISPR-based dual-target detection, we evaluated the impact of different components on the reaction. With increasing crRNA concentrations, the background fluorescence value increased (Fig. S2A and B), and the background-subtracted fluorescence value also slightly increased under the HEX channel of Cas12a detection (Fig. 3G), while little impact was observed under the FAM channel (Fig. 3F). Then, we tested concentrations of Cas13a protein and Cas12a protein. Both background fluorescence values slightly increased with increasing concentration (Fig. S2C and D). However, the background-subtracted fluorescence values decreased as the protein concentrations increased (Fig. 3H and J). In addition, the effectiveness of Cas13a detection was negatively impacted by increased Cas12a concentrations (Fig. 3I; Fig. S3A through F), and the background-subtracted fluorescence values of Cas12a were slightly influenced by Cas13a (Fig. 3J). These results suggest that there is mutual interference between Cas12a and Cas13a. With increasing concentrations of reporters, both background fluorescence values and background-subtracted fluorescence values increased, and no cross-reaction occurred (Fig. 3K and L; Fig. S2E and F). In addition to the above key components, the concentrations of T7 RNA polymerase and rNTP of the Cas13a system were also measured and had a slight inhibitory effect on Cas12a (Fig. S4A through D). Finally, we evaluated the optimal RPA reaction time in this system (we set 15, 30, 45, and 60 min) with samples of 10^3^ copies/μL and demonstrated that this detection assay can provide results within 75 min (15 min RPA with 60 min Cas12a/Cas13a reaction) (Fig. S4E and F). We thus optimized this CRISPR-based dual-target detection system according to the above tests and the final parameters can be acquired in Materials and Methods section (establishment of the CRISPR-based dual-target assay).

### Analytical sensitivity and specificity of CRISPR-based NG/CT detection

**Fig 4:**
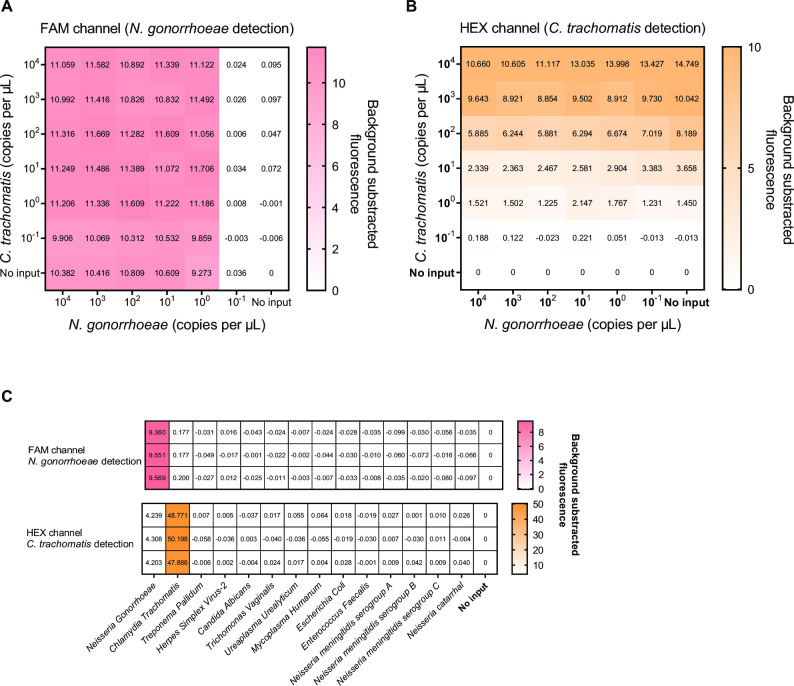
Sensitivity and specificity of the CRISPR-based dual-target detection assay. (**A and B**) The sensitivity of the CRISPR-based dual-target detection assay at various concentrations of the dsDNA templates of NG and CT, measured at the FAM and HEX channels. (**C**) Specificity of the CRISPR-based dual-target detection assay.

To evaluate the analytical sensitivity of the assay, we conducted an orthogonal experiment with synthetic dsDNAs (porA gene and cryptic plasmid fragments with concentrations ranging from 10^4^ to 10^−1^ copies/μL). This assay detected 10° copies/μL of both NG and CT in the FAM and HEX channels within 1 µL input DNA (Fig. 4A through B). Eight genital microorganisms with higher DNA concentrations and confirmed by TaqMan PCR (Fig. S5); no cross-reaction occurred between these samples in our assay (Fig. 4C). Therefore, the CRISPR-based dual-target detection system exhibited robust NG and CT detection.

### Performance of the CRISPR-based NG/CT detection system with clinical samples

To accurately evaluate the performance of PCR and CRISPR-based diagnostics, we compared our assay to in-house TaqMan PCR in parallel due to the larger volume of DNA used by the Roche Cobas 4800 assay relative to our new assay.

**Fig 5:**
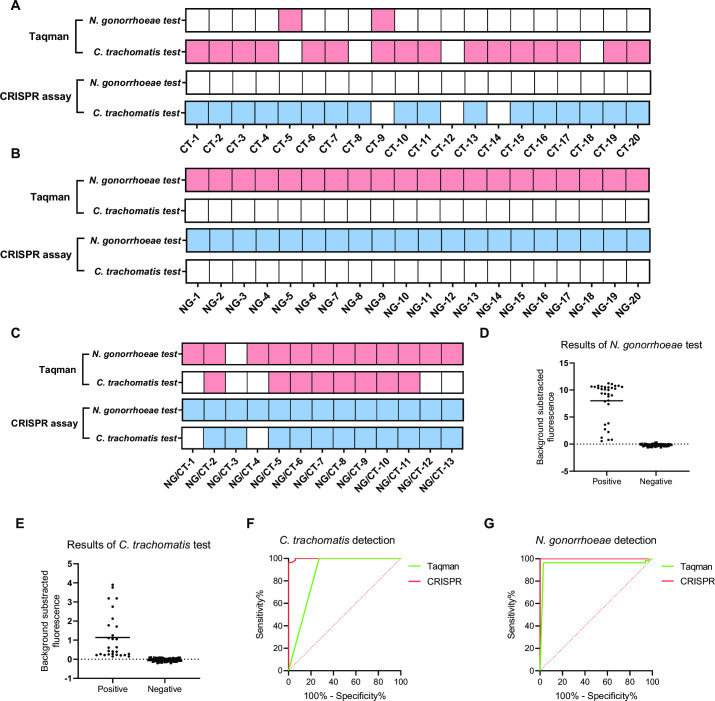
Evaluation of the CRISPR-based dual-target detection assay with clinical samples. (**A**) Results of the CRISPR-based dual-target detection assay and TaqMan PCR in clinical samples from patients with CT infection. (**B**) Results of the CRISPR-based dual-target detection assay and TaqMan PCR in clinical samples from patients with NG infection. (**C**) Results of the CRISPR-based dual-target detection assay and TaqMan PCR in clinical samples from coinfected patients. (**D and E**) Fluorescence value of the CRISPR-based dual-target detection assay, including positive and negative results, with all clinical samples in the FAM and HEX channels. (**F and G**) ROC curve of the CRISPR-based dual-target detection assay and TaqMan PCR in the detection of clinical samples.

The results showed that our assay did not falsely detect CT infection samples compared to in-house TaqMan PCR; our assay detected one additional sample (Fig. 5A). In the detection of NG infection samples, both methods can identify all specimens, and no false discrimination occurred (Fig. 5B). For coinfection samples, all samples were detected by CRISPR-based dual-target detection, except for two samples in which CT infection was not detected, and five clinical samples that TaqMan PCR failed to detect correctly (Fig. 5C). Our assay and TaqMan PCR were able to correctly categorize the 35 noninfected samples (Fig. S6A through D). To further evaluate the effectiveness of the CRISPR-based dual-target detection system, we collected fluorescence values of 88 clinical samples in the FAM and HEX channels. The fluorescence values of positive results in the FAM or HEX channel were based on receiver-operating characteristic (ROC) curves with an NG cutoff >0.31 and a CT cutoff >0.069 (Fig. 5D and E). The ROC curve also showed an area under the curve (AUC) of 0.99 and 1.00 for CT and NG detection compared to that of TaqMan PCR (0.86 and 0.95, respectively) (Fig. 5F and G). In the end, we tested our system in 12 clinical swab samples, which also showed good performance. In summary, the CRISPR-based dual-target detection system showed better detection of clinical samples and had higher PPA, NPA, and accuracy than TaqMan PCR (Table S2).

## DISCUSSION

We developed an isothermal dual-target detection assay that combines RPA amplification and Cas12a/Cas13a detection for the detection of NG and CT; the entire process can be carried out at 37°C. With optimal reaction conditions, this assay exhibited excellent performance in the detection of purified dsDNA templates as well as the detection of infection in 88 clinical samples from patients with known infection status.

Although traditional NAATs [e.g., PCR, ligase chain reaction, SDA, and TMA] have been developed to improve the diagnosis of STI, disadvantages such as high costs, limited performance, and restricted accessibility in resource-limited areas (19). Moreover, long turnaround times delay treatment and result in ongoing transmission of STI. The emergence of isothermal technology could help to solve the problems encountered in the traditional NAATs. Recently, a rapid and highly specific test for CT based on LAMP was developed with a detection limit of 4.5 × 10^3^ copies of CT *ompA* (36). However, LAMP is not suitable for detecting multiplex pathogens in patients with coinfections because four to six primers must be designed to recognize six distinct regions of the target sequences. RPA is another isothermal amplification assay; Zhai et al. utilized RPA for dual-target amplification of NG and CT based on RPA that used lateral flow detection to read the result (23), showing that the LOD only reached 200 plasmid copies per reaction. Neither of these isothermal amplification technologies can be robust to sensitivity, specificity, or dual target identification in NG/CT detection. Therefore, based on RPA detection, we further introduced the CRISPR assay by combining Cas12a and Cas13a in a single reaction to establish the CRISPR-based dual-target detection system. The major difference between our assay and other isothermal assays is that crRNA has high specificity due to its off-target effects resulting from mismatched base pairing, the T7 RNA polymerase can further amplify the RPA products, and the cleavage of RNA reporters by the collateral activity of Cas12a and Cas13a enhances the fluorescent signal and improves sensitivity. Hence, in the present study, our assay can achieve detection at the single molecular level of the dsDNA template, and it also shows good performance in sensitivity and specificity testing. To further test the performance of our assay in clinical samples, we wanted to be able to compare it with Roche Cobas 4800 dual-target test (CT/NG) which is a strictly verified commercial test used in the practice of clinical sample detection (37). Although the input volume of our assay is fivefold less than that of the Roche Cobas 4800 assay, our assay still achieves excellent PPA and NPA with the Roche Cobas 4800 assay (100% PPA and 100% NPA for NG, 84.85% PPA and 100% NPA for CT). In a parallel comparison, our assay achieved better accuracy than in-house TaqMan PCR. Thus, with these properties, we are better able to cope with the current increasing trends of NG/CT coinfection (2, 4).

Our approach has several limitations. First, our assay cannot operate in a single pot due to interference between the complicated reaction components of RPA and CRISPR detection (including various types of reagents, e.g., CutSmart Buffer, rNTPs Mix, Murine RNase inhibitor, T7 RNA Polymerase, etc.). Second, we did not evaluate the performance of our assays with other types of specimens, such as urogenital swabs. Third, fluorescence measurement instruments are currently required for our assay; however, in the future, this assay may be conducted in an instrument-free setting by utilizing lateral flow strips under optimization. Fourth, DNA extraction of clinical samples also needs to be simplified.

In conclusion, we successfully developed a CRISPR-based dual-target detection system that provides simultaneous detection of NG/CT. This isothermal assay provides excellent performance compared to in-house PCR and previously developed isothermal methods for NG/CT detection.

## SUPPLEMENTAL MATERIAL

Fig S1 spectrum.00464-23-s0001.docx Supplemental figures.

TABLE S1 spectrum.00464-23-s0002.xlsx Sequence of each primer used in this study.

TABLE S2 spectrum.00464-23-s0003.xlsx Detection results of clinical samples.

ASM does not own the copyrights to Supplemental Material that may be linked to, or accessed through, an article. The authors have granted ASM a non-exclusive, world-wide license to publish the Supplemental Material files. Please contact the corresponding author directly for reuse.
