# Supplementary material for: An isothermal CRISPR-based diagnostic assay for Neisseria gonorrhoeae and Chlamydia trachomatis detection
Source: Microbiol Spectr. 2023 Oct 26;11(6):e00464-23. doi: 10.1128/spectrum.00464-23 (PMC10715037; doi:10.1128/spectrum.00464-23)
Supplement: Fig S1 — Supplemental figures. [file spectrum.00464-23-s0001.docx]

**Figure S1. Validation of multiplex RPA for NG and CT amplification.** Multiplex RPA can amplify the NG and CT targets in a single tube.

**
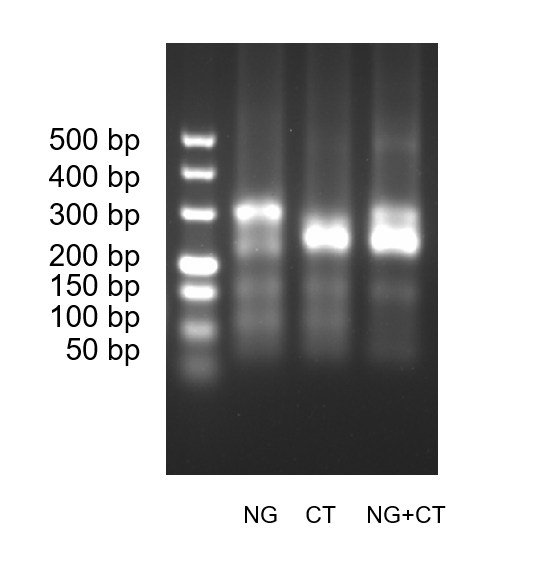
**

**Figure S2. Background fluorescence of each tested key component of the CRISPR detection system. A-B** Background fluorescence of this assay given various concentrations of Cas13a crRNA and Cas12a crRNA, measured at the FAM and HEX channels. **C-D** Background fluorescence of this assay given various concentrations of Cas13a protein and Cas12a protein, measured at the FAM and HEX channels. **E-F** Background fluorescence of this assay given various concentrations of reporters, measured at the FAM and HEX channels.

**A**

**B**

**C**

**D**

**E**

**F**

**Figure S3. Kinetics measurement of the impact of Cas12a concentration on the CRISPR detection system. A-F** Increased concentrations of the Cas12a protein have a negative effect on the Cas13a protein.

**
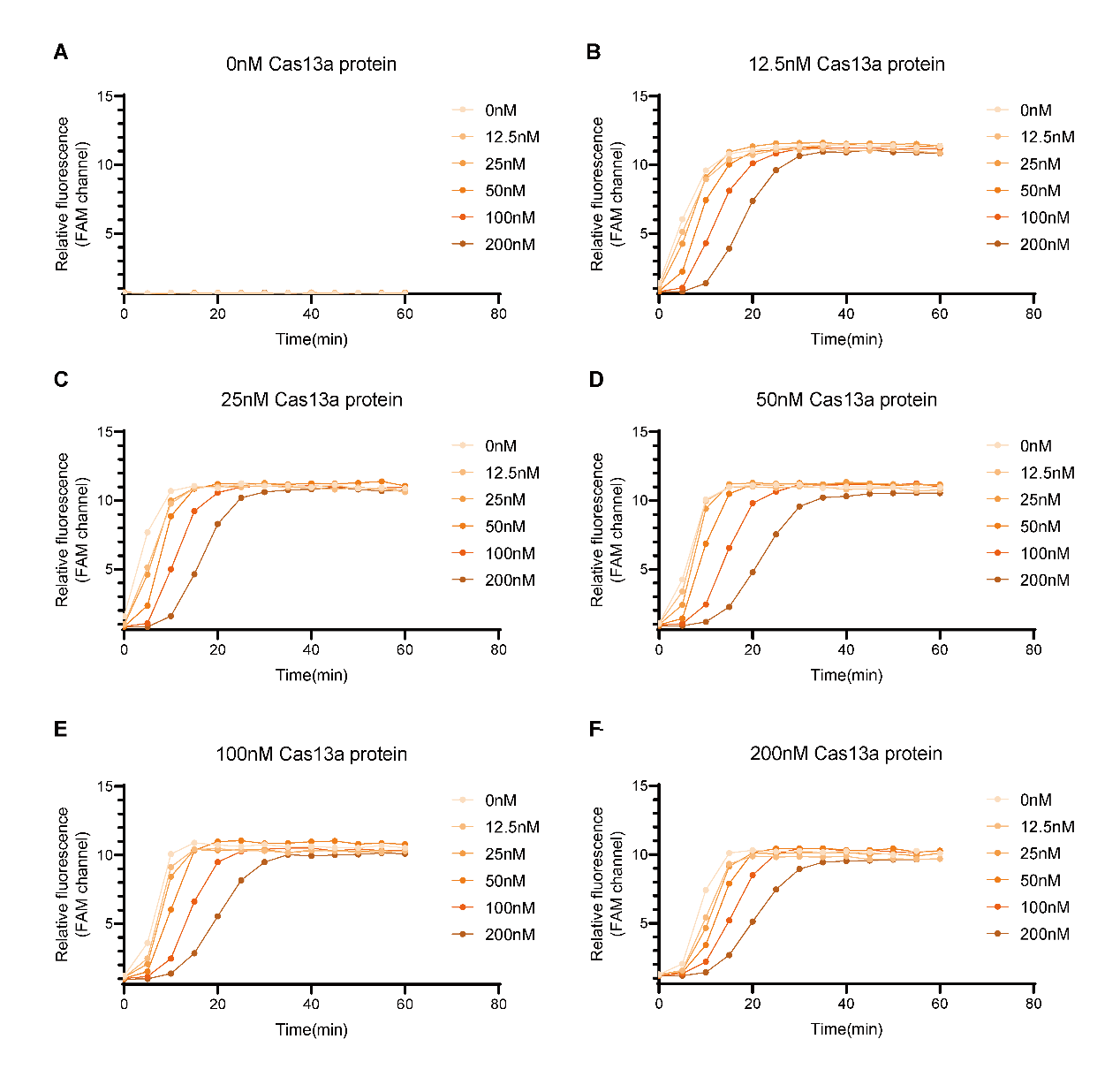
**

**Figure S4. Evaluating the impact of other parameters in the** **CRISPR-based dual-target detection system. A-B** The effect of T7 RNA polymerase in this assay. **C-D** The effect of rNTP in this assay. **E-F** Fluorescence measurement of the CRISPR-based dual-target detection system with different RPA amplification times.

**
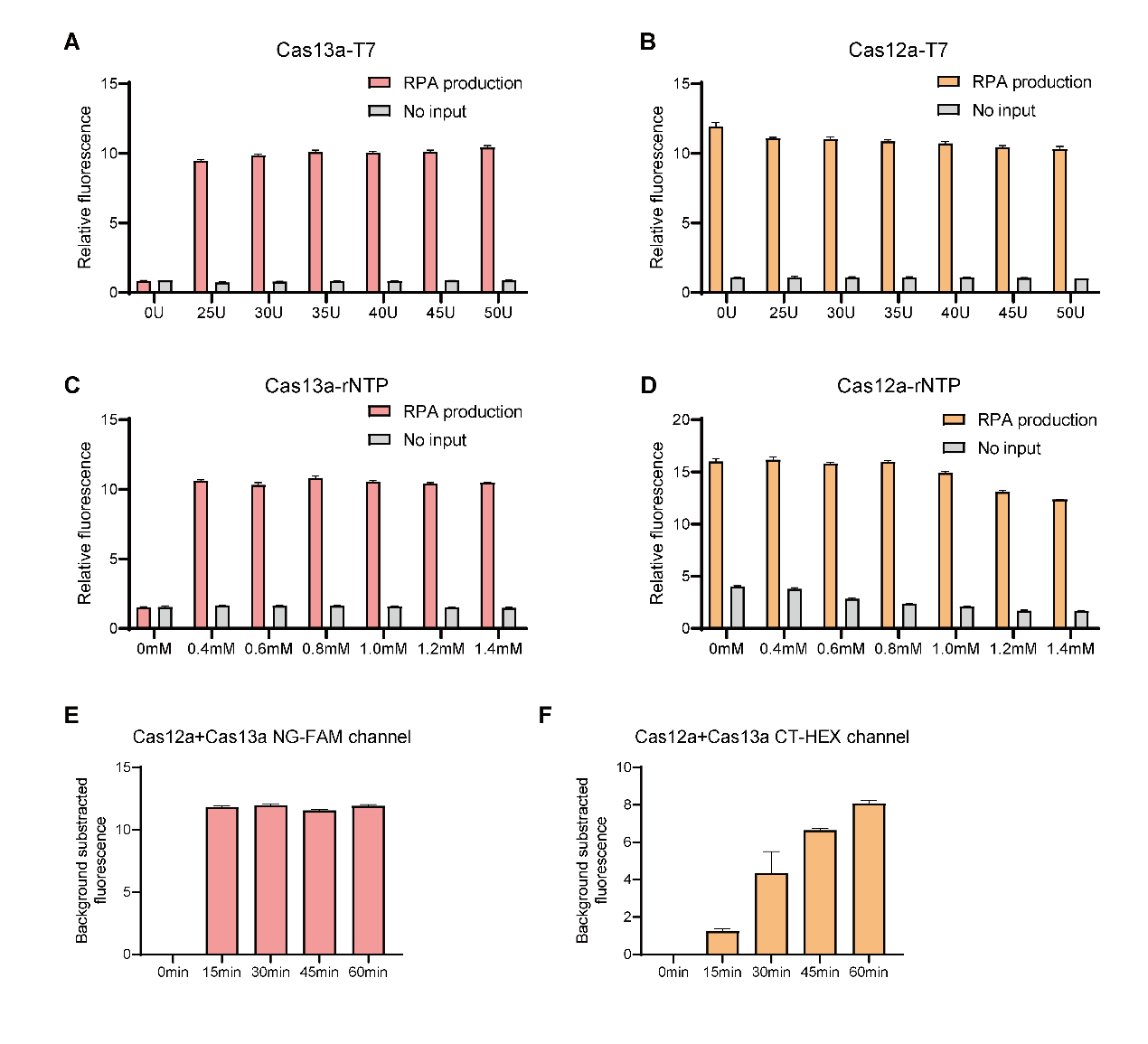
**

**Figure S5. TaqMan PCR validation of 8 genital microorganisms.** Cycle threshold (CT) value of TaqMan PCR for 8 genital microorganisms.

**Figure S6. Results of the CRISPR-based dual-target detection assay and TaqMan PCR in clinical negative samples. A-D** The results of TaqMan PCR and CRISPR-based dual-target detection assays in 35 clinical samples collected from noninfected people.

**B**

**A**

**C**

**D**

**Figure S7. Results of the CRISPR-based dual-target detection assay in clinical swab samples.** Fluorescence value of CRISPR-based dual-target detection assay for 12 clinical swab samples.
